# 

**Published:** 1879-05

**Authors:** 


					﻿Whole Number,
CCCCVIII.
May, 1879.
Number 5,
Vol. XXXVIII.
THE
CHICAGC
MEDICAL
J ournal and Examiner
EDITORS:
WILLIAM H. BYFORD, A.M., M.D.,
JAS. NEVINS HYDE, A.M., M.D, FERD. C. HOTZ, M.D.
E. FLETCHER INCALS, M.D.
CHICAGO:
PUBLISHED BY THE CHICAGO MEDICAL PRESS ASSOCIATION.
188 Clark Street.
^'Read the Notice of this Journal among Advertisements.
				

